# Detecting anthropogenic footprints in sea level rise

**DOI:** 10.1038/ncomms8849

**Published:** 2015-07-29

**Authors:** Sönke Dangendorf, Marta Marcos, Alfred Müller, Eduardo Zorita, Riccardo Riva, Kevin Berk, Jürgen Jensen

**Affiliations:** 1 Department of Civil Engineering, Research Institute for Water and Environment, University of Siegen, Paul-Bonatz-Strasse 9-11, 57076 Siegen, Germany; 2 IMEDEA (UIB-CSIC), Miquel Marquès, 21, E-07190 Esporles, Spain; 3 Department of Mathematics, University of Siegen, Walter-Flex-Strasse 3, 57072 Siegen, Germany; 4 Helmholtz-Centre Geesthacht, Max-Planck-Strasse 1, 21502 Geesthacht, Germany; 5 Departement of Geoscience and Remote Sensing, Delft University of Technology, Stevinweg 1, 2628 Delft, Netherlands

## Abstract

While there is scientific consensus that global and local mean sea level (GMSL and LMSL) has risen since the late nineteenth century, the relative contribution of natural and anthropogenic forcing remains unclear. Here we provide a probabilistic upper range of long-term persistent natural GMSL/LMSL variability (*P*=0.99), which in turn, determines the minimum/maximum anthropogenic contribution since 1900. To account for different spectral characteristics of various contributing processes, we separate LMSL into two components: a slowly varying volumetric component and a more rapidly changing atmospheric component. We find that the persistence of slow natural volumetric changes is underestimated in records where transient atmospheric processes dominate the spectrum. This leads to a local underestimation of possible natural trends of up to ∼1 mm per year erroneously enhancing the significance of anthropogenic footprints. The GMSL, however, remains unaffected by such biases. On the basis of a model assessment of the separate components, we conclude that it is virtually certain (*P*=0.99) that at least 45% of the observed increase in GMSL is of anthropogenic origin.

## Abstract (web summary)

The contribution of anthropogenic forcing to rising sea levels during the industrial era remains uncertain. Here, the authors provide a probabilistic evaluation and show that at least 45% of global mean sea level rise is of anthropogenic origin.

## Graphical abstract

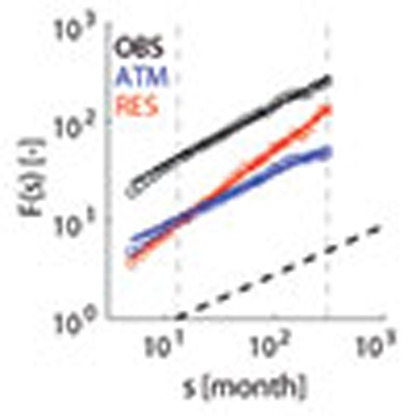

Tide gauge records along coastlines provide evidence that mean sea levels (MSLs) have risen since the late nineteenth century with globally averaged rates of 1.33–1.98 mm per year^1^,^2^,^3^,^4^. These rates derived from instrumental records are larger than the value reported for the past 2,000 years using indirect proxy estimators^5^,^6^. A question that remains is how much of this rise is related to past and current natural climate variations or to anthropogenic forcing. Answering this question is challenging, since our measurements available over the past two centuries represent only a very narrow window of the Earth's history, limiting our knowledge about natural low-frequency signals in the climate system.

Understanding the persistence of natural variations is a fundamental prerequisite to distinguish between natural and anthropogenic causes of the contemporary global MSL (GMSL) and local MSL (LMSL) rise. Knowing the persistence of naturally forced GMSL and LMSL change, in turn, enables a statistical assessment of the probability that the recent rise is outside the range of natural variability, and more robust estimates of the uncertainty in (and therefore the significance of) observed trends. While in most sea level investigations it has been assumed that the persistence of GMSL and LMSL change diminishes after a few months to years^1^,^2^,^3^,^4^, recent works demonstrate that there is also persistence on timescales of decades^7^,^8^. This persistence, which can be accurately described by the Hurst exponent *α* (see Methods), leads naturally to multi-decadal excursions from the mean state and can therefore produce relatively strong multi-decadal to centennial trends unrelated to any external forcing^7^,^8^. On the basis of the assumption that the natural variability can be accurately modelled by a purely long-term correlated process, it was recently suggested that more than 50% of the observed twentieth-century LMSL rise (and more than 58% of the GMSL rise) can be attributed to external causes unrelated to natural climate variations^8^ (thus including an anthropogenic contribution and local human interventions or vertical land motion). However, this assumption still needs to be verified considering that MSL is an integrative measure representing the cumulative response of the ocean to multiple physical processes^9^ with diverse spectral properties^7^.

While the two main contributors to the recent GMSL rise (thermal expansion and the melting of land-locked ice^9^) are, beside the possible anthropogenic long-term trend, governed by slow natural variations with Hurst exponents *α* larger than 0.8 (that is, strong long-term correlations)^7^, the major drivers of LMSL change (ocean redistribution of heat, salt and water mass in response to changing winds, ocean circulation and air–sea fluxes^10^,^11^,^12^) were found to decay exponentially, thus rather resembling a short-term correlated process^7^. Therefore, the processes contributing to LMSL changes act on various timescales, leading to different spectral properties. This further raises the question whether one statistical model is able to reproduce these different properties.

Here we revisit the characteristics of natural variability in LMSL and GMSL by assessing the spectral properties of different components. We demonstrate that in some regions the high-frequency variability due to atmospheric forcing masks intrinsic low-frequency variability related to volumetric MSL changes (density and barystatic mass changes and their redistribution). These masking effects lead locally and regionally to an underestimation of the true natural variability and, consequently, to an erroneously enhanced significance of the observed twentieth-century LMSL rise. This finding is underpinned by Monte-Carlo experiments with time series where the spectral properties are *a priori* known. Since the masking effect is mainly related to wind-driven redistribution processes, the GMSL, however, remains unaffected. We provide even with an improved estimate of natural GMSL variability unambiguous evidence for a significant anthropogenic footprint in GMSL rise since 1900.

## Results

### Spectral properties of LMSL

**Figure 1:**
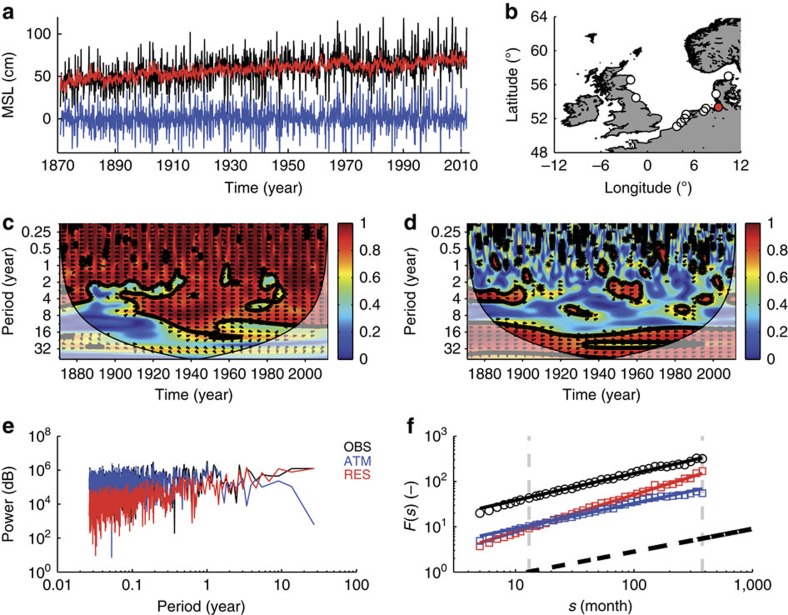
Spectral properties of LMSL and its components. (**a**) Time series of OBS (black), ATM (blue) and RES (red) at the tide gauge of Cuxhaven over the period from 1871 to 2011. The location of the tide gauges is shown in (**b**) with Cuxhaven marked in red. Wavelet coherence plots (Morlet wavelet) of ATM and RES versus OBS are shown in (**c**,**d**). The black lines show the 5% significance level using the red noise model. The corresponding global power spectra for OBS, ATM and RES inferred from a fast Fourier transformation are shown in (**e**,**f**). Fluctuation functions estimated with DFA2 for OBS, ATM and RES, respectively. The grey dotted lines mark the time window (13≤*s*≤423 months) for which the Hurst exponents *α* were estimated. The black dotted line marks a Hurst exponent *α* of 0.5, that is, uncorrelated noise.

We base our approach on established techniques^8^,^13^,^14^,^15^,^16^ and estimate the upper range of possible naturally forced centennial trends in LMSL and GMSL for a given confidence level (*P*=0.99) and the resulting minimum contribution related to external causes (here we concentrate only on the linear contribution that can be produced by external/natural forcing). This is achieved by computing the probability of the occurrence of naturally forced centennial trends with the statistical parameters derived from a second-order detrended fluctuation analysis (DFA2) or standard autoregressive models of the order one (AR1; see Methods). From the distribution of natural trends, one can directly obtain the significance *S* of an observed trend as well as the minimum external contribution unrelated to any natural fluctuation (for a given level of confidence). This minimum external contribution is defined as the difference between the observed trend and the 99th percentile of the distribution of natural trends^8^,^13^,^14^,^15^ (Methods and Supplementary Fig. 1). These rather conservative confidence bounds are used, since we search for the upper bounds of natural variability. Results for the respective 95% confidence intervals can be found in Supplementary Figs 5–6 and Supplementary Table 1. Unlike in previous works^8^,^13^,^14^,^15^ we categorize the observed LMSL (OBS) in two components and assess their statistical properties separately: a quickly acting atmospheric component (ATM) and a more slowly varying residual component (RES; difference between OBS and ATM), comprising the volumetric contributions from density and mass changes and their redistribution. Focusing on 11 centennial (seasonally adjusted) tide gauge records (see Methods and Table 1) from the North Sea (Fig. 1b), we estimate the ATM at each location using the multiple linear regression model (LRM) from ref. 11. The LRM identifies relevant predictors from a pool of atmospheric reanalysis data (zonal/meridional winds and sea level pressure (SLP) from the twentieth century reanalysis^17^ in an area of ±2° around the respective tide gauge) using a stepwise algorithm with forward selection^10^,^11^. The model has been successfully validated against a state-of-the art barotropic hydrodynamical model (Hamburg Shelf Ocean Model: HAMSOM^18^) for the second half of the twentieth century^11^ (see also Methods and Supplementary Fig. 2) and is used here to model the ATM over the period 1871–2011.

To better understand the temporal characteristics of ATM and RES compared with OBS, all contributions (Fig. 1a) are assessed for the tide gauge of Cuxhaven in the non-stationary (Fig. 1c,d) and stationary spectral power space (Fig. 1e). The two components ATM and RES show different timescale characteristics. While the ATM displays its largest spectral power on timescales below a few years, the spectral power of the RES increases with frequency becoming largest at low frequencies (Fig. 1e). This leads to a dominant coherence between ATM and OBS at timescales shorter than a decade (Fig. 1c), whereas longer variations are closely linked to RES (Fig. 1d). In other words, rapid MSL variations in Cuxhaven (and in the North Sea in general, see Supplementary Fig. 3) are mostly associated with barotropic processes in response to the atmospheric forcing^11^,^19^, while decadal fluctuations are mostly related to persistent volumetric changes and their redistribution^7^.

Given their different spectral characteristics, DFA2 is applied separately to OBS, its ATM and RES at each site. Figure 1f shows the fluctuation functions for Cuxhaven (those for the other sites can be found in the Supplementary Fig. 4). The slope *α* of the fluctuation function (equivalent to the Hurst exponent *α*) was computed between 13 and 423 months (∼35 years; Fig. 1e). The fluctuation function of the ATM steadily increases with *α* close to 0.5, an indicator that these processes have characteristics similar to white or AR1 noise. In contrast, the analysis of OBS reveals the presence of long-term correlations, with Hurst exponents *α* of 0.6. Once the ATM is removed from observations *α* significantly increases at all stations (0.8–0.9 for RES, Table 1). This is in line with ref. 7 who noted that semi-enclosed basins and continental shelves display smaller Hurst exponents than large oceanic basins, where the contribution of atmospheric forcing is smaller and damped by the advection of heat leading to a reddening of the spectra^20^. We thus conclude that the presence of rapidly varying short-term processes, such as those generated by the atmospheric forcing over shallow shelves, mask the long-term correlation in MSL records driven by slow and persistent processes, such as gradual MSL rise due to ocean thermal expansion or mass exchange and its redistribution^9^,^10^.

### Short-term noise and the estimation of natural LMSL trends

**Figure 2:**
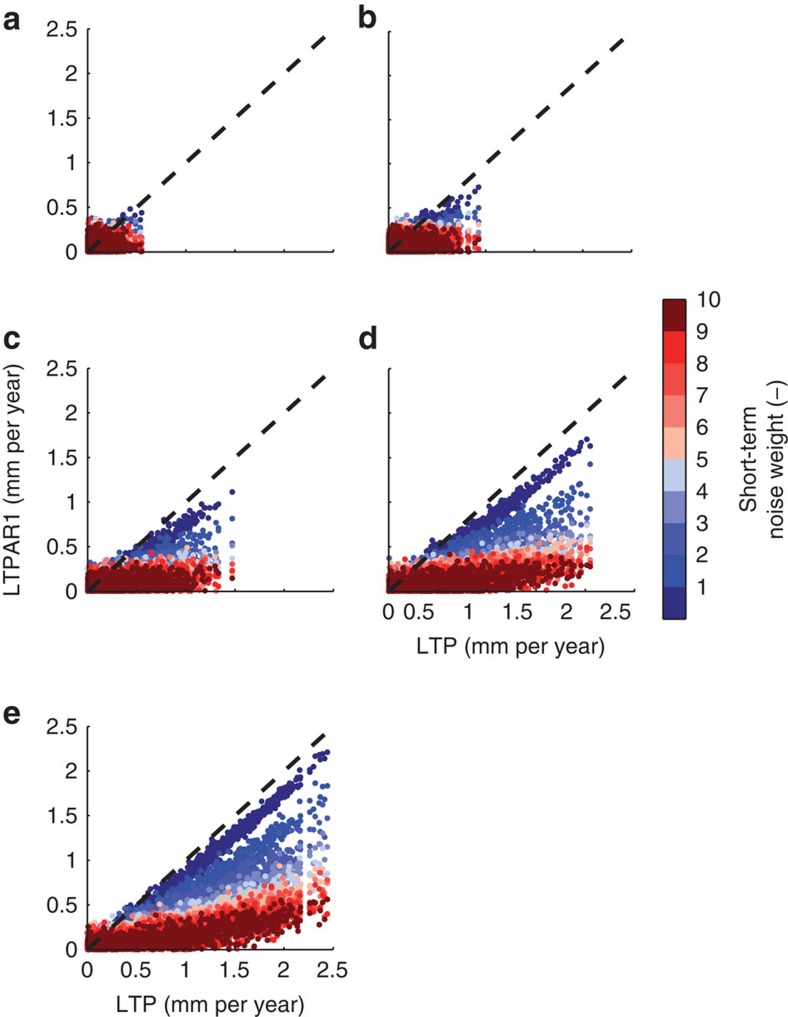
The effect of AR1 noise on modelling natural and external trends in LMSL. (**a**–**e**) Natural trends in long-term correlated Gaussian data (LTP; 1,000 time series with a length of 1,692 months) versus natural trends in long-term correlated Gaussian data with additive AR1 noise (LTPAR1). The different colour shades correspond to the weight with which the AR1 noise was added to the long-term correlated data (1–10 times larger than the s.d. of the purely long-term correlated Gaussian data; in the North Sea the s.d. of the ATM exceeds that of the RES by 1–3 times). The natural trends correspond to a time series with a unit s.d. of 10 cm in the combined time series (which is roughly the median in the analysed North Sea records). Simulation results are shown for long-term correlated Gaussian data with (**a**) *α*=0.6, (**b**) *α*=0.7, (**c**) *α*=0.8, (**d**) *α*=0.9 and (**e**) *α*=1.0. For the lag-1 autocorrelation a value of *c*=0.1, which is the maximum value obtained from the ATM, has been applied.

The inability to estimate the actual long-term correlation in presence of short-term correlated processes implies that the natural variability of a given time series may not be properly described^21^. Consequently, the distribution of trends associated with natural variability can be biased, with large implications for the determination of the statistical significance of observed trends as well as their minimum external contribution. This is underpinned by a Monte-Carlo experiment (Fig. 2a–e), where the distributions of natural trends in a set of long-term correlated data are compared in cases with and without additive AR1 noise. Different degrees of long-term correlations have been tested, with Hurst exponents between 0.6 and 1.0, as well as different AR1 noise weights having a s.d. 1–10 times larger than the purely long-term correlated time series (note that the weight between ATM and RES ranges in the North Sea from 0.6 to 3 depending on the site). It is obvious that for small Hurst exponents (Fig. 2a, *α*=0.6), which are close to the theoretical value of white or AR1 noise (*α*=0.5), the distribution of the associated trends does not change when AR1 noise (with such small lag-1 autocorrelations as obtained from ATM) is added. However, with increasing *α*, the presence of noise induces an underestimation of natural trends reaching 0.4–0.5 mm per year for centennial records with Hurst exponents comparable to those inferred from the RES in the North Sea (Table 1, Fig. 1c,d).

**Figure 3:**
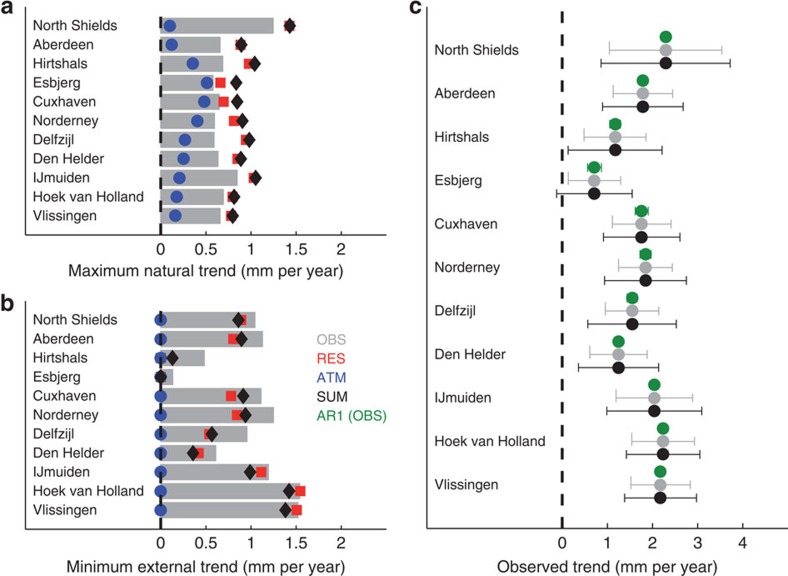
Maximum natural and minimum/maximum external trends in LMSL at tide gauges in the North Sea. (**a**) Maximum natural trends (*P*=0.99) derived by the method from refs 9, 13, 15 for Hurst exponents *α* estimated with OBS (grey bars), ATM (blue dots), RES (red squares) and the root sum of squares of ATM and RES (SUM, black diamonds) over the period from 1900 to 2011. The corresponding minimum external trends, calculated as the difference between the observed and maximum natural trends, are shown in (**b**). In (**c**) the observed trends are shown in its classical expression with their lower and upper 99% confidence bounds (that is, the minimum and maximum external contribution, see also Methods) obtained from OBS (grey) and SUM (black). For comparison also the results for a classical AR1 model are shown (green). Note that the observed trends still contain vertical land motions, which are responsible for the vast majority of the differences obtained between the different stations.

Applying the separate calculation of natural and minimum external trends to the 11 tide gauge records in the North Sea basin (Fig. 3a,b) leads to estimates that are in close agreement to those inferred from the Monte-Carlo experiment (Fig. 2a–e). The sum (here, the root sum of squares) of natural trends derived by a separate assessment of ATM and RES exceeds those applying the integrated approach^8^ by up to 0.4 mm per year on a centennial timescale from 1900 to 2011 (Fig. 3a), which corresponds to roughly one-fourth of the entire twentieth-century LMSL rise. When the ATM is subtracted, the long-term correlation of the LMSL record is better captured (increase of *α*) and the magnitude of the trend associated to natural variations is increased by an amount of up to 0.4 mm per year. In consequence, the significance of the observed trends is reduced (Table 1) and the minimum fraction of the observed local trends related to external causes is smaller (Fig. 3b), implying that previous attempts^8^ have likely overestimated the minimum amount of anthropogenic forcing in LMSL rise. It is also important to notice that the resulting confidence bounds of the observed trends are even up to four times larger than in the classical approach where AR1 noise has been assumed (Fig. 3c and Supplementary Fig. 5)^1^,^2^,^3^,^4^.

**Figure 4:**
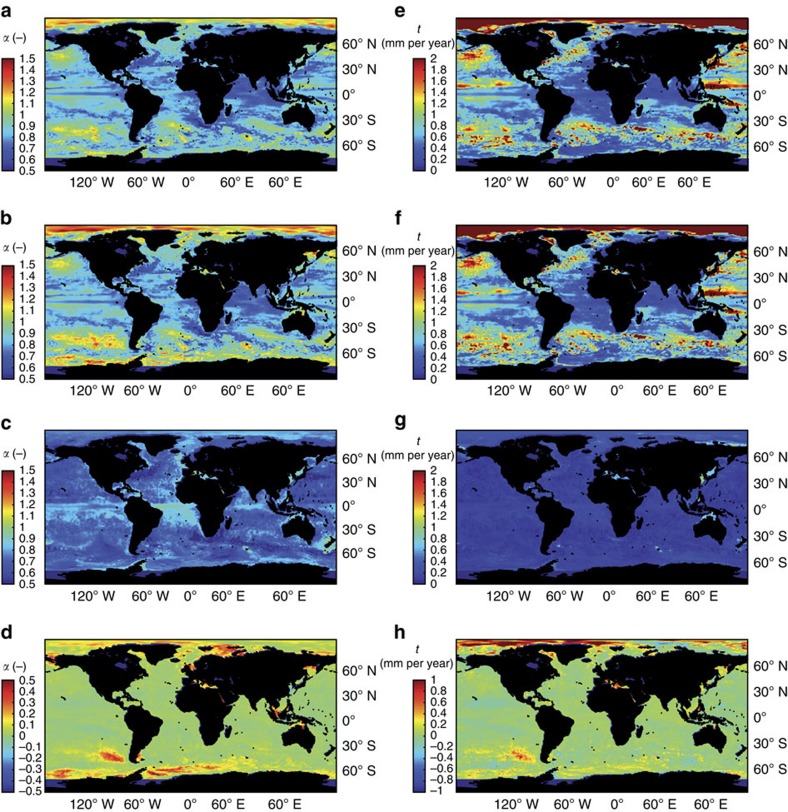
Hurst exponents *α* and maximum natural centennial trends in modelled LMSLsyn. (**a**–**c**) *α* values as calculated for different components of LMSLsyn, RESsyn and OBPsyn over the period from 1899 to 2008. (**d**) Differences between the *α* values from LMSLsyn and RESsyn. (**e**–**g**) Maximum natural trends (*P*=0.99) for LMSLsyn, RESsyn and OBPsyn fields under the assumption of a short-term (*α*=0.5) or a long-term correlated (*α*>0.5) process. (**h**) Differences between maximum natural trends in sea level derived from an integrated assessment of LMSLsyn minus the root sum of squares of natural trends calculated for OBPsyn and RESsyn separately.

An assessment on a global scale is currently hampered by the absence of reliable long-term estimates of the ATM contribution. Instead, we use synthetically modelled LMSL (LMSLsyn) fields as a combination of the dynamic sea-surface height (SSH) from a centennial ocean reanalysis^22^, the barystatic contribution resulting from the mass loss of 18 glacier regions^23^, the Greenland ice sheet^24^ and their corresponding fingerprints^25^ as well as variations in land hydrology^26^ (see Methods). We separate the SSH from the ocean reanalysis into the contributions from the local steric height and ocean bottom pressure (OBPsyn) variations and combine the local steric height with the fingerprints from glaciers and ice sheets as well as hydrology to a volumetric component RESsyn. Although OBP still contains non-local steric variations^22^, the evaluations provide a first approximation of ATM and therefore for the bias induced by not properly describing the spectrum of LMSL. We calculated the Hurst exponents *α* (Fig. 4a–c) and the resulting natural trends for the LMSLsyn, the OBPsyn and the RESsyn (Fig. 4e,f and Supplementary Fig. 6). In large parts of the world oceans the long-term correlations are confined to the RESsyn (Fig. 4b), while the OBPsyn signal exhibits Hurst coefficients close to AR1 noise (Fig. 4c). This leads to an underestimation of the Hurst exponent *α* present in RESsyn (Fig. 4d). As expected, significant differences are found between natural trends calculated within an integrated and a separate assessment (Fig. 4h). These differences are largest in areas where OBP changes are known to dominate the LMSL variations^27^,^28^,^29^ (for example, South Pacific, Mediterranean Sea and Arctic Ocean). In such dynamically active regions naturally forced centennial trends of LMSLsyn tend to be underrated by an amount of up to 1.0 mm per year or even larger (Arctic Ocean), demonstrating the importance of accounting for the spectral diversity of different processes involved into LMSL variations.

### Natural and external trends in GMSL

Since the differences found in the naturally forced centennial trends are mostly related to the masking effect of wind-driven redistribution processes, they do not have any effect on GMSL trends and their significance. GMSL variations are driven by barystatic mass and density changes^1^,^9^, both of which are strongly long-term correlated^7^. Hence, naturally forced centennial trends can be assessed for the GMSL using the integrated assessment by applying the DFA2 for the estimation of the Hurst exponent *α* to the original GMSL record. However, a major problem of recent GMSL reconstructions^2^,^3^,^4^ is that while they all agree in the sign of their long-term trends^1^,^2^,^3^,^4^,^26^, their temporal variability remains highly uncertain with respect to the true global mean^26^. For instance, the variability in the Church and White^2^ (CW11) reconstruction mostly reflects LMSL variations measured by tide gauges along the coast rather than the true GMSL variations^26^, whereas the Hay *et al*.^4^ (H15) reconstruction only represents a smoothed signal without any inter-annual variability. The temporal variability in the Jevrejeva *et al*.^3^ (J14) reconstruction, by contrast, is significantly overestimated compared with the true global mean (Supplementary Fig. 7) and becomes increasingly uncertain back in time due to the sparse tide gauge network in the early twentieth century^3^. Consequently, an accurate description of naturally forced centennial trends with these time series^8^ is not possible. To overcome the sampling problem of sparse tide gauge records in the earlier part of the twentieth century, we form a new synthetic GMSL curve (GMSLsyn) from the spatially homogeneous LSMLsyn fields. On inter-annual and longer timescales, this GMSLsyn curve is significantly correlated with the observed GMSL from altimetry (∼0.5) and shows a considerable smaller s.d. (although still slightly overestimated) than the other reconstructions^2^,^3^ over the altimetry period since 1993 (Supplementary Fig. 7).

**Figure 5:**
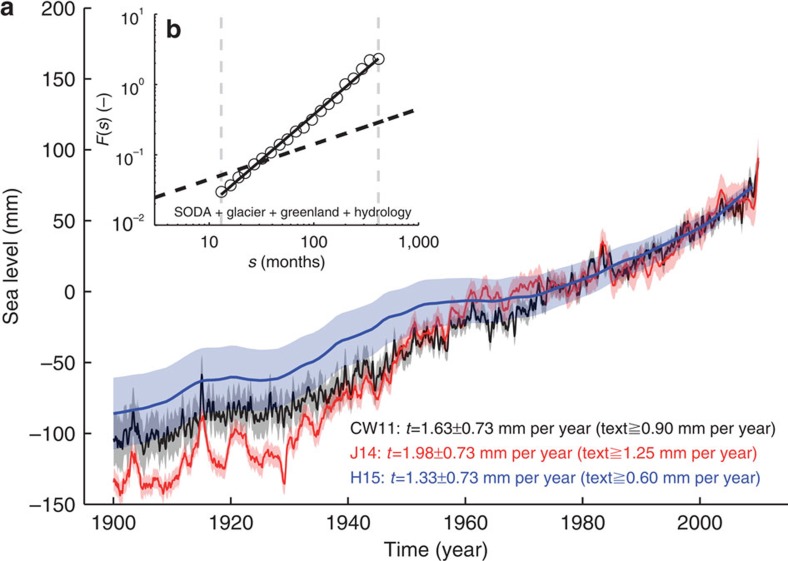
Maximum natural and minimum external trends in GMSL. (**a**) Shown are three recent reconstructions of GMSL based on CW11 (ref. 2), J14 (ref. 3) and H15 (ref. 4) over the period 1900–2009. The shadings show their 1*σ* uncertainties. Their linear trends are given in the legend. All reconstructions suffer, with respect to their temporal variability, from sampling problems related to the temporally and spatially unevenly distributed location of tide gauge measurements^23^. Therefore, the temporal GMSL variability and the resulting naturally forced centennial trends (*P*=0.99) have been assessed using spatial average of the LMSLsyn fields (SSH, glacier, Greenland ice sheet and hydrology), which are available over the entire global ocean over the period from 1871 to 2008. The resulting fluctuation function derived from a DFA2 is shown in (**b**). The fluctuation function yields an *α* value of 1.28. Following the approach described in refs 8, 13, 14, 15 this implies an upper bound of naturally forced centennial trends (1900–2009) of 0.73 mm per year (*P*=0.99). This suggests that the observed twentieth century GMSL rise is already outside the range of natural variability with a minimum external contribution (dependent on the reconstruction) of 0.60–1.25 mm per year (*P*=0.99).

We search for the presence of long-term correlations in the GMSLsyn curve using DFA2 (Fig. 5a,b), which yields a Hurst exponent *α* in the order of 1.28 (Fig. 5b). In combination with a s.d. of 9.1 mm over the entire twentieth century this leads to maximum naturally forced centennial trends of 0.73 mm per year (*P*=0.99), 0.52 mm per year (*P*=0.95) and 0.43 mm per year (*P*=0.90) for the period 1900–2009. These values are significantly larger than previous estimates^1^,^2^,^3^,^4^, where the presence of long-term correlations was neglected. However, even with this improved estimate all twentieth-century GMSL reconstructions^2^,^3^,^4^ point towards a significant (*S*>0.99) long-term trend, which cannot be solely explained by natural variability.

## Discussion

The results demonstrate that the presence of short-term processes impacts significantly on the determination of the trends associated with natural variations in LMSL time series (up to 1 mm per year on a centennial scale). The removal of the ATM is therefore essential for a correct description of the natural variability of long-term processes, especially in those areas where the ATM contribution accounts for a large part of the total LMSL variance, as is the case in most shallow continental shelf seas^19^. When this natural variability is properly characterized, it permits a more sophisticated statistical judgment of the significance of observed trends and therefore a quantification of the minimum fraction that can be attributed to an external forcing unrelated to natural climate long-term variations. In the North Sea, 10 out of 11 stations show a significant long-term trend (*S*>0.99) leading to minimum external trends (*P*=0.99) that range between 0.13 and 1.42 mm per year (Fig. 3b and Table 1). These values should not be interpreted simply as the contribution of a common external forcing because an external trend in a single record may well be due to local effects such as measurement errors (as it is probably the case in North Shields (Fig. 3a,b)) or vertical land movements due to glacial isostatic adjustment or local subsidence/uplift, and they refer to the minimum value, meaning that the actual external contribution could be much larger but masked by natural variability.

On a global scale the short-term fluctuations of wind-driven redistribution processes cancel out and the resulting GMSL changes are suggested to represent a hybrid of long-term correlated natural variability (Hurst exponent *α* of 1.28) and relatively smooth anthropogenic forcing^7^,^8^,^30^. Accounting for the long-term correlated structure of GMSL variations yields an upper range of possible natural centennial trends in the order of 0.73 mm per year since 1900. Glacial isostatic adjustment corrected local and regional late Holocene proxy evidence shows rates that do not exceed this value^5^,^6^,^31^. Considering that the GMSL variability in observations and model simulations is at least an order of magnitude less than the local/regional variability^30^, our findings are therefore consistent with the paleo evidence from periods in which the anthropogenic contribution was absent. Comparing our model-based estimate of natural GMSL variability with the observed twentieth-century GMSL rise of 1.33–1.98 mm per year^1^,^2^,^3^,^4^ therefore suggests that it is virtually certain (*P*=0.99) that at least 45% (1.33–0.73 mm per year) of the observed twentieth-century GMSL rise is of anthropogenic origin, extremely likely (*P*=0.95) that it is at least 61% (1.33–0.52 mm per year) and very likely (*P*=0.90) that it is at least 68% (1.33–0.43  mm per year). Likewise, our estimate of possible naturally forced centennial trends enhances the upper bound of the possible anthropogenic contribution. Hence, it is also virtually certain (*P*=0.99) that the anthropogenic contribution does not exceed a value of 2.71 mm per year (1.98+0.73 mm per year), extremely likely (*P*=0.95) that it does not exceed 2.50 mm per year (1.98+0.52 mm per year) and very likely (*P*=0.90) that it does not exceed 2.41 mm per year (1.98+0.43 mm per year). The majority of the external contribution is probably related to the two dominant contributors to global MSL rise over the twentieth century^1^, that is, glacier melting and thermosteric MSL, both of which became increasingly determined by anthropogenic forcing during the second half of the twentieth century^32^,^33^,^34^.

## Methods

### Tide gauge data

Eleven centennial tide gauge records of monthly resolution from the North Sea basin were selected for this study (Supplementary Table 1 and Fig. 1b). Nine of the records were downloaded from the website of the Permanent Service for Mean Sea Level^35^, while the other two records (Cuxhaven, Norderney) stem from the database published by ref. 36. The reason for limiting the analyses of tide gauge data to this particular region is that reliable long-term estimates for the ATM are freely available only here^11^. For our assessment we remove the mean seasonal cycle from each record by subtracting the average of each calendar month taken over the entirely available period. No corrections for the effects of vertical land motion have been applied, since these values are still very scarce^37^ and models for glacial isostatic adjustment very uncertain^3^.

### Modelled MSL data

In addition to the tide gauge records, we built model-based LMSLsyn fields on a global scale by adding together recent reconstructions of the dynamic SSH, the glacier contribution from 18 major mass loss regions and the Greenland ice sheet combined with the respective fingerprints, and temporally varying land hydrology. The contributions from the Antarctic ice sheet is expected to be of minor importance over the twentieth century^38^,^39^ and therefore neglected in the present study. For the dynamic SSH the Simple Ocean Data Assimilation (SODA) reanalysis^22^ over the period from 1871–2008 is used. SODA is a global baroclinic ocean reanalysis forced with winds from the twentieth-century reanalysis^17^ that assimilates temperature and salinity observations^22^. Since SODA is based on the Boussinesq approximation, we accounted for missing steric effects following the approach introduced by ref. 40: that is, adding a globally uniform average of the steric height at each grid point and time step. For completeness we also added the missing long-term trend to SODA using the CMIP5 historical ensemble trend of the global mean thermosteric component over the period 1901–2000 (0.47 mm per year) as published by ref. 38. The good performance of SODA with respect to inter-annual and decadal LMSL variability has already been demonstrated^7^,^26^ and is therefore suggested to be of sufficient quality for our purposes.

The contribution of glacier melting and the Greenland ice sheet is incorporated at each grid point by multiplying the sea level contribution as reconstructed by refs 23, 24 with the respective fingerprints^25^. Glacier fingerprints are computed by solving the sea level equation^41^, which accounts for the gravitational coupling between surface redistribution of water masses and deformation of the solid earth, on a compressible elastic earth and including the effect of changes in earth rotation^42^.

Despite these two factors, which determined the majority of the long-term trends in twentieth-century GMSL^1^,^38^,^39^, hydrology is known to be responsible for a large fraction of the observed inter-annual GMSL variability^43^. We follow ref. 26 and produce an estimate of this contribution under the assumption that it acts as a barotropic load on the ocean, that is, monthly soil moisture fields from the Climate Prediction Centre^44^ are used to model hydrology changes as a globally uniform but temporally varying signal in the form of:


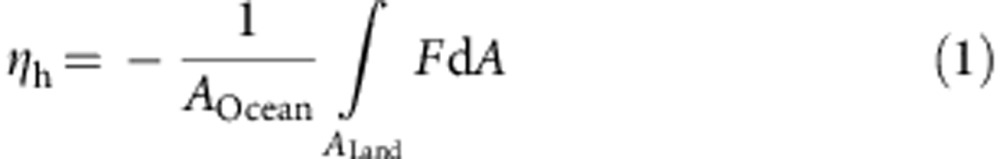


where *A*_Ocean_ and *A*_Land_ are the surface area of the ocean and the continents, respectively, and *F*, the detrended soil moisture^26^. Unfortunately, the soil moisture component is only available from 1948 on. To artificially extend this time series back in time to 1871 we apply a phase-randomized Fourier algorithm^45^. The algorithm is based on a Fourier transformation of the original time series and a randomization of the phases at different frequencies in the range of 0 and 2*π*. This allows for an artificial extension of the existing record back to 1871 showing a similar spectrum and autocorrelation function as the observations. Although this time series does not provide the truly observed signal before 1948, it gives a reasonable estimate of the underlying processes with a similar temporal characteristic^45^.

To separate the contributions from local steric and OBP variations from the dynamic SSH in SODA the common nonlinear equation of the state of the ocean has been applied to estimate the local steric height. The OBPsyn component was derived by simply removing the local steric height from the model SSH at each grid point. The modelled volumetric component RESsyn is consequently derived as the sum of the steric height from SODA, the glacier and ice sheet contribution and hydrology at each time step and grid point, while OBPsyn serves as a proxy for the ATM.

### Determination of the ATM

The determination of the ATM is based on a multiple stepwise LRM introduced by refs 11, 46. For the LRM, SLP and zonal and meridional wind stress time series showing the highest bivariate correlation with the observed MSL from an area of ±4° around the tide gauge location are selected as independent forcing factors. Monthly detrended MSL anomalies from the respective tide gauge location are then used as the dependent variable, while the monthly detrended SLP and wind stress anomalies are taken as independent predictors. The stepwise algorithm chosen in the LRM is based on a forward selection where one by one the predictors showing the highest correlation with the MSL anomalies are included into the model. The inclusion of variables is stopped when one of the obtained regression coefficients gets insignificant (95% confidence level using common *F*-statistics). To estimate the contribution of the ATM to long-term trends, we assume that the relationship found for the detrended records also holds on longer timescales and simply combine the estimated regression coefficients with the non-detrended atmospheric forcing variables. By definition, ATM and RES are uncorrelated.

The performance of this model has been intensively tested in ref. 11. However, to provide an overview about the performance for the 11 selected stations from this study, we validate the model against a state-of-the art barotropic hydrodynamical HAMSOM model of the North Sea introduced by ref. 18. The HAMSOM model is driven by the eight main tidal constituents at the model boundaries and 10 m surface winds from reanalysis data sets and has a spatial resolution of 3 km^18^. For the validation both models, that is, the LRM as well as HAMSOM, have been forced with atmospheric fields from the National Centers for Environmental Prediction reanalysis^47^. The HAMSOM runs are available over the period 1953–2003. Hence, we limit the validation to this period. The results are shown in Supplementary Fig. 2. For 9 out of 11 tide gauge locations the estimated ATMs show reasonable agreement between the two different models as underpinned by correlation coefficients larger than 0.9. Only two stations display somewhat smaller correlations coefficients in the order of 0.6–0.8. These smaller values are probably related to local bathymetric inaccuracies in the numerical HAMSOM model rather than a poor performance of the LRM as pointed out in ref. 11. Overall, the validation demonstrates the ability of the LRM in capturing the barotropic ATM in the North Sea with sufficient accuracy, which underpins the applicability for the purposes of this study.

### Estimation of natural and external trends

To estimate a relative MSL trend over a specific time period *L*, we apply a common linear least-squares-fit to the monthly MSL anomalies, which gives us an absolute change Δ (that is, the difference between the last and the first value of the regression line) and the s.d. *σ* of the residuals around the regression line. Following refs 8, 13, 14, 15 we consider the relative trend ratio *x=*Δ*/σ* as the quantity of interest. A central question regarding the observed MSL change is now whether *x* is natural or anthropogenic in origin. Therefore, we require the probability *P*(*x; L*)d*x* that in time series with a similar autocorrelation structure as the observed data, a relative trend between *x* an *x+*d*x* occurs. From *P* we can derive the exceedance probability


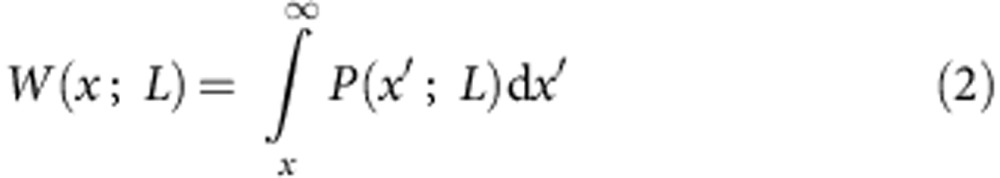


and the respective significance


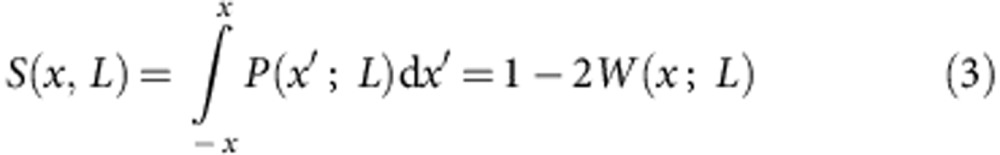


of *x*. By definition, *S* is the probability that the relative trend in the record lies between *–x* and *x* (refs 8, 13, 14, 15).

Here we assume that an observed trend *x* cannot be explained by natural variability alone, if *S* is above 0.99 (*P*=0.99). Consequently, relative trends *x* between *–x*_99_(*L*) and *x*_99_(*L*) are considered as natural. If *x*_99_ is exceeded in an observational record, the part *x*−*x*_99_ cannot be explained by natural variations alone. Thus, *x*−*x*_99_ must be externally forced and can be considered as the minimum external trend. Furthermore, we also know that the external trend cannot exceed a value of *x*+*x*_99_, which therefore appears to be the maximum possible external trend^15^.

It has been shown in extensive Monte-Carlo experiments^15^ that for records of sufficient length *L* the probability density function *P*(*x*; *L*) is distributed in many models as Student's *t*, which can be written as


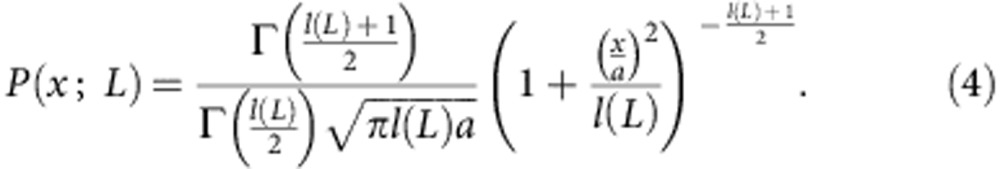


*P*(*x*; *L*) is therefore a function of the scaling parameter *a* and the effective length *l*, both of which depend on the autocorrelation structure of the observed record. For instance, if a record is only characterized by short-term correlations with a specific lag-1 autocorrelation *c* one can easily obtain *l* and *a* by following the approach of ref. 48: that is,


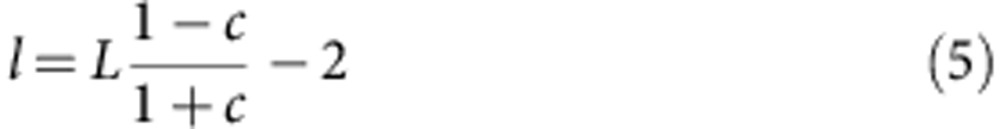


and


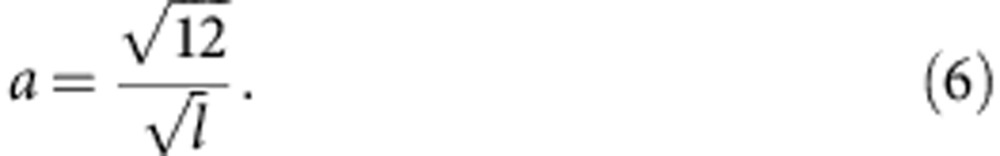


However, as recently shown^7^,^8^, this assumption might not be appropriate with respect to the fact that MSL records also exhibit long-term correlations characterized by a Hurst exponent *α*>0.5. In these cases tables for *a* and *l* based on extensive Monte-Carlo simulations have been provided in the appendix of ref. 15. To decide which case is appropriate in our assessment, we calculate the lag-1 autocorrelation as well as the Hurst exponent *α* using DFA2 (ref. 49). DFA2 has been established especially for the case of non-stationary time series and is very robust against external trends in a given record^49^. DFA2 integrates a time series to a random walk series *Y*(*t*) (by calculating the cumulative sum), separates the random walk series in non-overlapping segments, each of them containing *s* points, removes quadratic trends from each segment, calculates the variance *Y*_s_ for each segment, takes the average over all segments, and finally computes the root mean square to obtain a fluctuation function *F*(*s*):


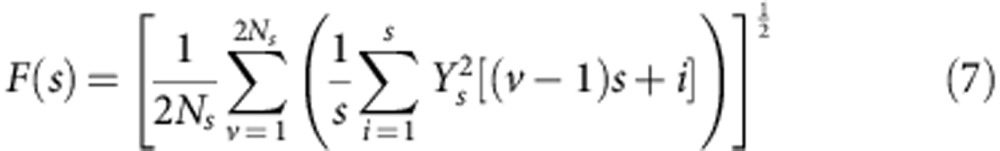


The Hurst exponent *α* can be estimated as the asymptotic slope of *F*(*s*) in the double logarithmic space. A time series is long-term correlated if *α* is significantly different from 0.5 and short-term correlated or uncorrelated if *α* is equal to 0.5. If we find significant long-term correlations we consequently derive *l* and *a* from the tables in ref. 15, whereby in case of a Hurst exponent *α* smaller or equal 0.5, *l* and *a* are calculated following ref. 48. Further information about the general mathematical concept of estimating natural and external trends can be found in the literature^13^,^14^,^15^.

When dividing the observed sea level signal into the two separate components ATM and RES we estimate the quantiles of naturally forced centennial trends based on two separate *t*-distributions with different scaling parameters *a* and effective length *l*. In case of a normal distribution and uncorrelated records of ATM and RES one can easily obtain approximations of the combined quantiles via a common root sum of squares:


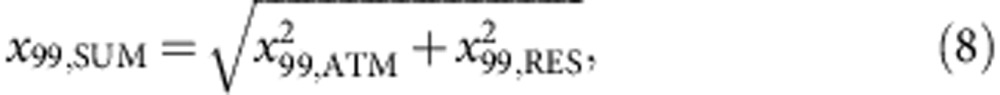


while in case of significant correlations between RES and ATM the quantiles can be calculated as:


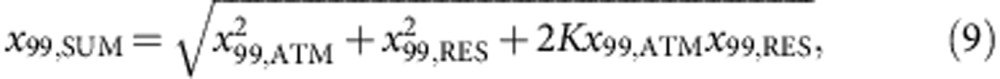


where *K* represents the linear correlation between ATM and RES. However, the natural trends are obtained from Student's *t*-distributions for which the accuracy of the root sum of squares approximation is not known. Therefore, we have tested whether the approximations from equations (8) and (9) also hold for Student's *t*-distributions with similar characteristics as obtained from RES and ATM. As shown in Supplementary Fig. 8 this is still the case. Note that significant correlations between RES (RESsyn) and ATM (OBPsyn) have been only found in the Arctic Ocean and some smaller sub-areas with minor influences on the calculation of the naturally forced trends (Supplementary Fig. 9). For the North Sea tide gauges all correlations between ATM and RES are zero (not shown).

## Additional information

**How to cite this article:** Dangendorf, S. *et al*. Detecting anthropogenic footprints in sea level rise. *Nat. Commun.* 6:7849 doi: 10.1038/ncomms8849 (2015).

## Supplementary Material

Supplementary Information Supplementary Figures 1-9, Supplementary Table 1 and Supplementary Reference

## Figures and Tables

**Table 1:** Results from the estimation of natural and external trends in SLR.

**Station**	**Availability**	**Hurst exponent** ***α***			**LAG1 autocorrelation** ***c***			**Observed trend (mm per year) and significance**			**Natural trend (mm per year)**		**External trend (mm per year)**	
		**OBS**	**ATM**	**RES**	**OBS**	**ATM**	**RES**	**OBS**	Case 1*	**Case 2**†	**Case 1**	**Case 2**	**Case 1**	**Case 2**
Vlissingen	1900–2011	0.72	0.52	0.86	0.14	0.01	0.51	2.18	(1.00)	(1.00)	0.66	0.80	1.52	1.38
Hoek van Holland	1900–2011	0.71	0.51	0.86	0.17	0.00	0.45	2.24	(1.00)	(1.00)	0.69	0.81	1.54	1.42
Ijmuiden	1900–2011	0.73	0.52	0.90	0.16	0.01	0.51	2.04	(1.00)	(1.00)	0.85	1.05	1.19	0.99
Den Helder	1900–2011	0.67	0.54	0.89	0.13	0.06	0.45	1.25	(1.00)	(0.99)	0.64	0.89	0.61	0.36
Delfzijl	1900–2011	0.62	0.51	0.86	0.13	0.04	0.40	1.55	(1.00)	(0.99)	0.59	0.98	0.96	0.57
Norderney	1900–2011	0.61	0.56	0.84	0.15	0.11	0.50	1.84	(1.00)	(1.00)	0.60	0.91	1.25	0.94
Cuxhaven	1900–2011	0.60	0.56	0.82	0.16	0.11	0.50	1.76	(1.00)	(0.99)	0.65	0.85	1.11	0.91
Esbjerg	1900–2011	0.57	0.56	0.80	0.15	0.11	0.43	0.71	(1.00)	(0.81)	0.58	0.84	0.13	−
Hirtshals	1900–2011	0.65	0.56	0.86	0.20	0.14	0.37	1.17	(1.00)	(0.99)	0.69	1.04	0.48	0.13
Aberdeen	1900–2011	0.73	0.50	0.88	0.21	0.05	0.39	1.79	(1.00)	(1.00)	0.66	0.89	1.13	0.89
North Shields	1900–2011	0.88	0.52	0.96	0.35	0.04	0.53	2.29	(1.00)	(1.00)	1.25	1.43	1.05	0.86

The table provides an overview over the main values derived from an assessment of each tide gauge record in the North Sea. The Hurst exponents *α* are derived from a DFA2. All trends are calculated for the common period from 1900 to 2011 using a confidence level of 99% (*P*=0.99). The significance *S* of each trend is provided in brackets.

^*^Case 1: integrated assessment.

^†^Case 2: separate assessment.
