# Supplementary material for: Detecting anthropogenic footprints in sea level rise
Source: Nat Commun. 2015 Jul 29;6:7849. doi: 10.1038/ncomms8849 (PMC4532851; doi:10.1038/ncomms8849)
Supplement: Supplementary Information — Supplementary Figures 1-9, Supplementary Table 1 and Supplementary Reference [file ncomms8849-s1.pdf]

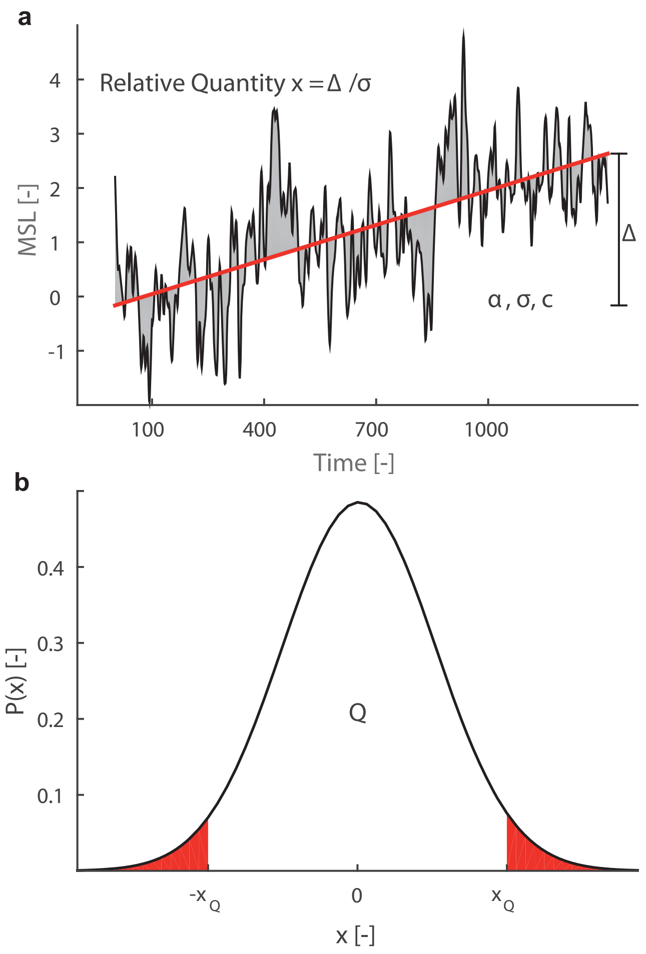

Supplementary Figure 1 | Estimating natural trends in MSL. a, Visualization of the quantities relative trend $x=\Delta/\sigma$, standard deviation σ, and the autocorrelation value (depending on the case: Hurst exponent α or lag-1 autocorrelation c) for a synthetic data set of the length L = 1320 months. Δ is the total linear long-term change as determined by a common least squares fit (red line). σ is the standard deviation of the residuals (grey) around the target signal. The Hurst exponent α or lag-1 autocorrelation c describes the memory in the data set. The quantity of interest is the ratio $x=\Delta/\sigma$. **b,** Probability density function of natural trends $x=\Delta/\sigma$. The integral over the white area below the curve defines the confidence probability Q. The confidence interval is given by $-x_Q$ and x_Q. If an observed trend is outside this interval, it is considered to be unnatural.

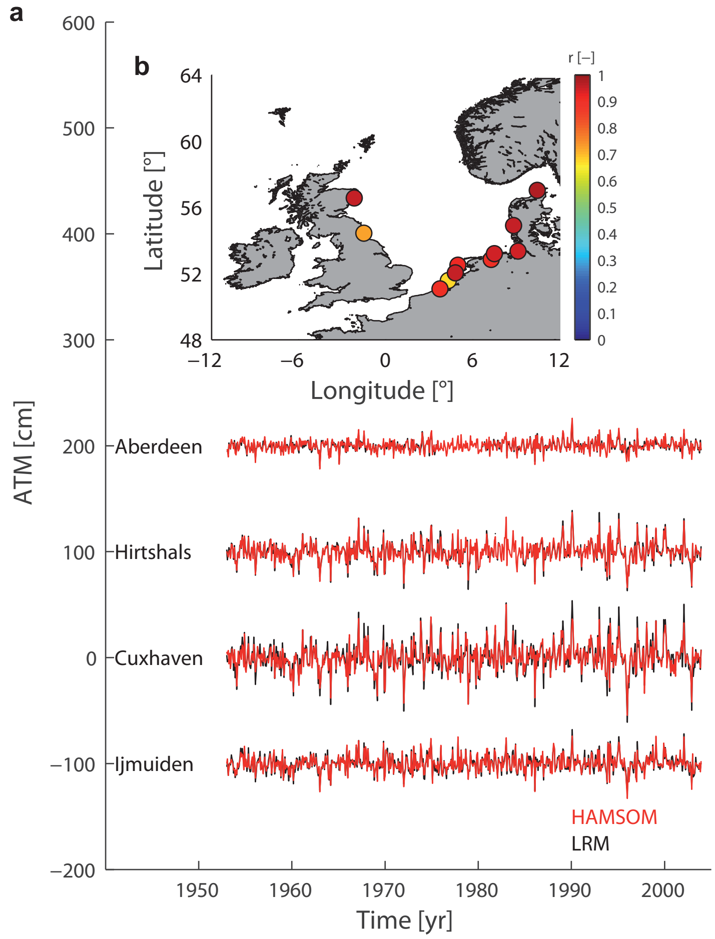

Supplementary Figure 2 | Performance of the LRM. Shown is a comparison between the ATM derived via the LRM and the state of the art numerical model HAMSOM. Four selective time series from around the North Sea are shown in **a**, while the correlation coefficients of all tide gauge locations are shown in **b**.

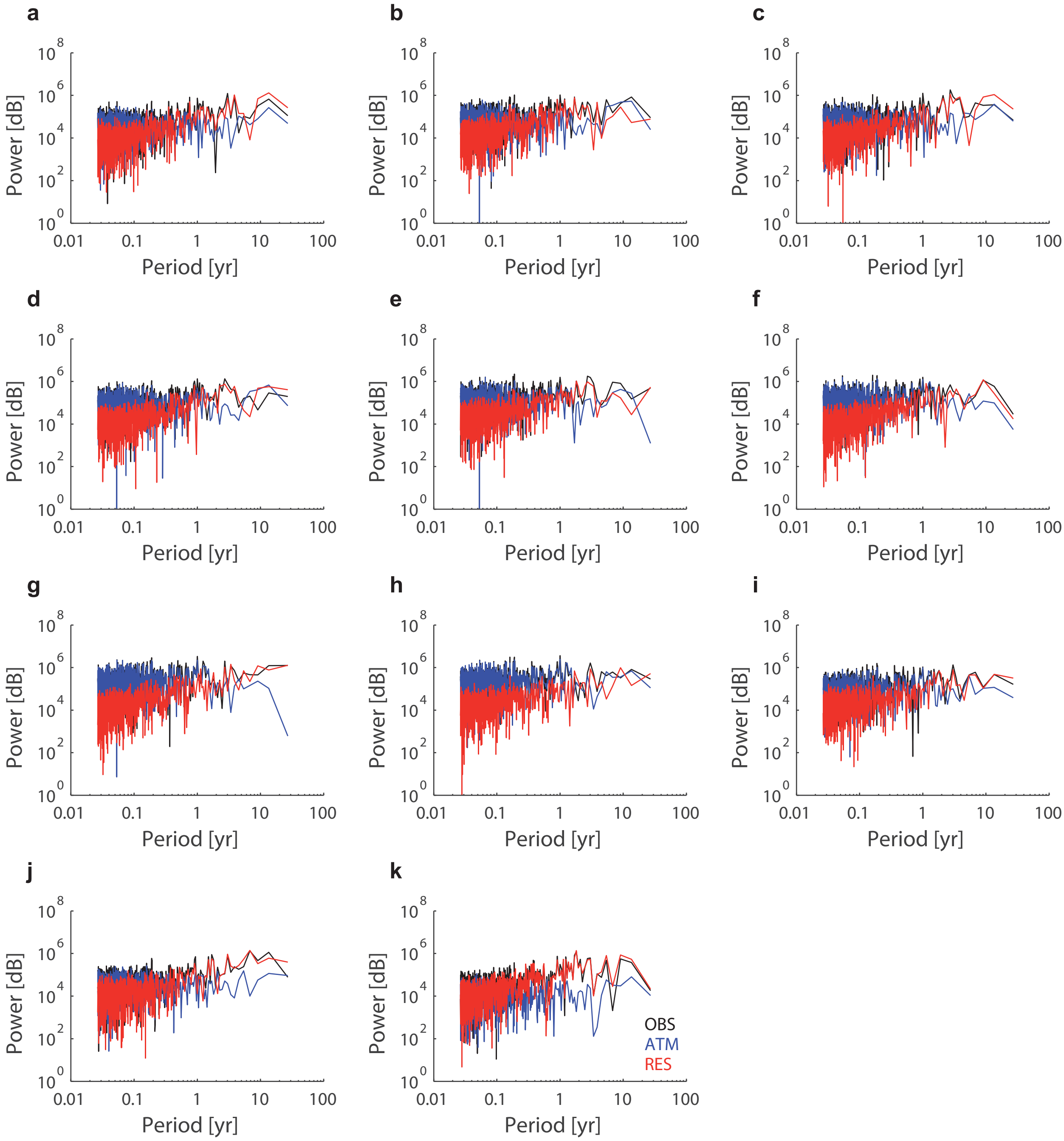

Supplementary Figure 3 | Power spectra of MSL time series and its components. Global power spectra for OBS (black), ATM (blue) and RES (red) from a Fast Fourier Transformation for the tide gauges at **a,** Vlissingen, **b,** Hoek van Holland, **c,** Ijmuiden, **d,** Den Helder, **e,** Delfzijl, **f,** Norderney, **g,** Cuxhaven, **h,** Esbjerg, **i,** Hirtshals, **j,** Aberdeen, and **k,** North Shields. Data gaps have been filled via linear multiple regressions with neighboring stations.

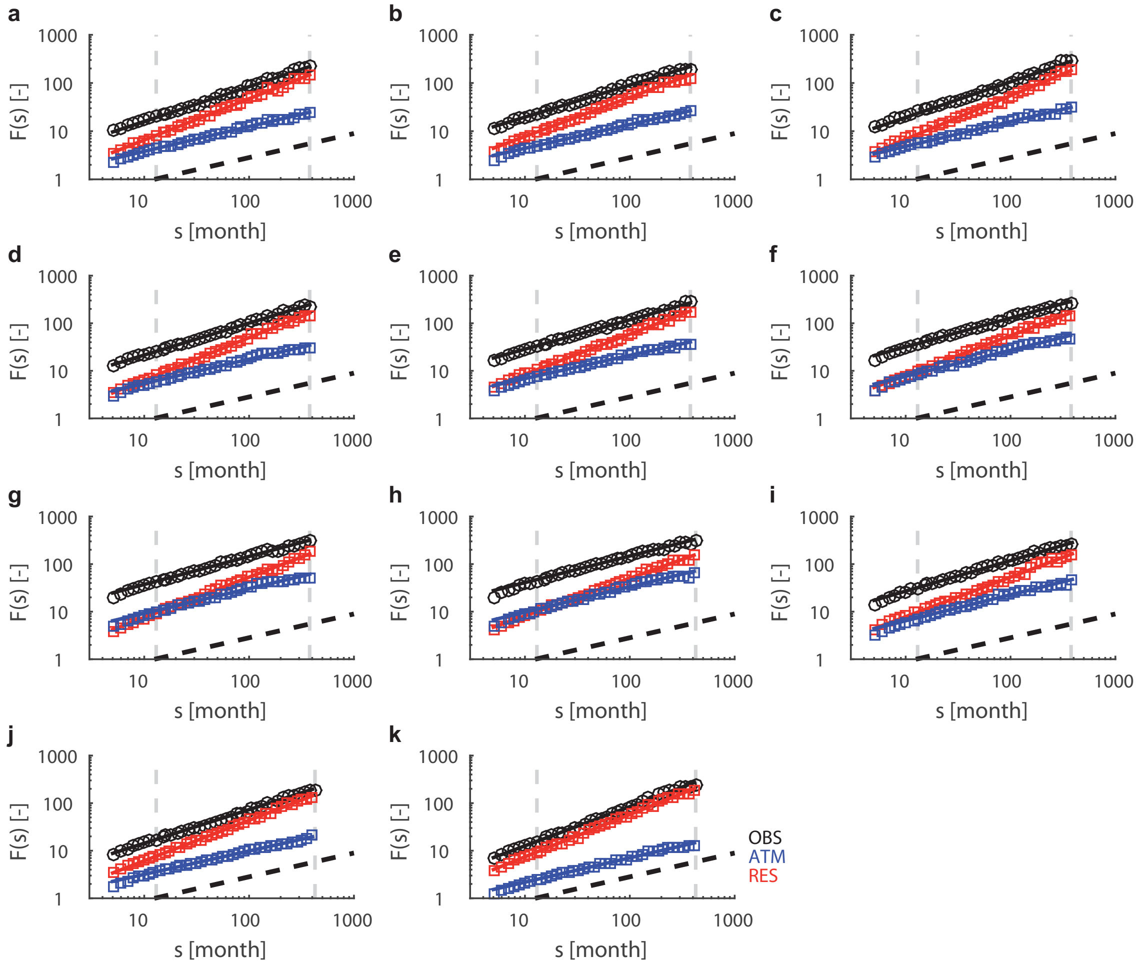

Supplementary Figure 4 | DFA2 of MSL time series and its components. Fluctuations functions derived from a DFA2 for OBS (black), ATM (blue) and RES (red) for the tide gauges at **a,** Vlissingen, **b,** Hoek van Holland, **c,** Ijmuiden, **d,** Den Helder, **e,** Delfzijl, **f,** Norderney, **g,** Cuxhaven, **h,** Esbjerg, **i,** Hirtshals, **j,** Aberdeen, and **k,** North Shields. The grey dotted lines mark the time window (13<=s<=423 months) for which the Hurst exponents α were estimated. The black dotted line marks a Hurst exponent α of 0.5, i.e. uncorrelated noise.

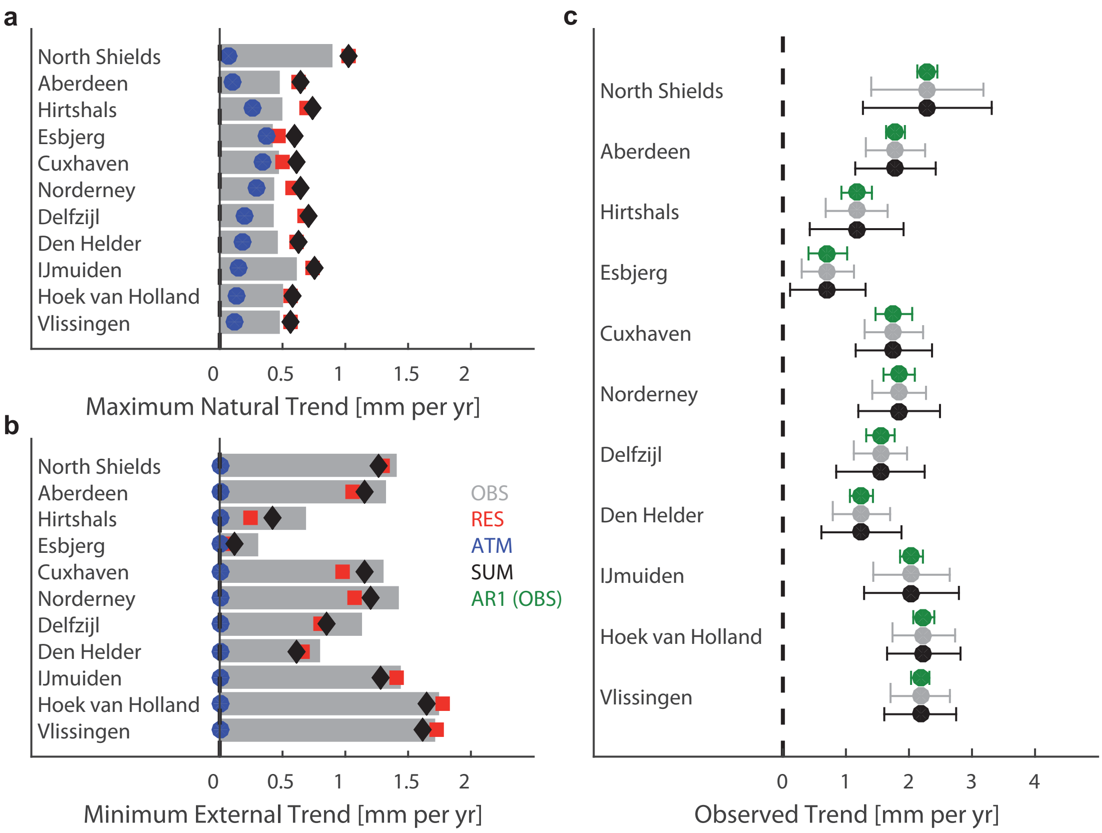

Supplementary Figure 5 | Maximum natural and minimum/maximum external trends in LMSL at tide gauges in the North Sea. a, Maximum natural trends (P=95) for Hurst exponents α estimated with OBS (grey bars), ATM (blue dots), RES (red squares), and the sum of ATM and RES (SUM, black diamonds) over the period 1900-2011. The corresponding minimum external trends, calculated as the difference between the observed and maximum natural trends are shown in **b**, respectively. In **c** the observed trends are shown in its classical expression with their lower and upper 95% confidence bounds (i.e. the minimum and maximum external contribution, see also **Methods**) obtained from OBS (grey) and SUM (black). For comparison also the results for a classical AR1 model are shown (green). Note that the observed trends still contain vertical land motions, which are responsible for the vast majority of the differences obtained between the different stations.

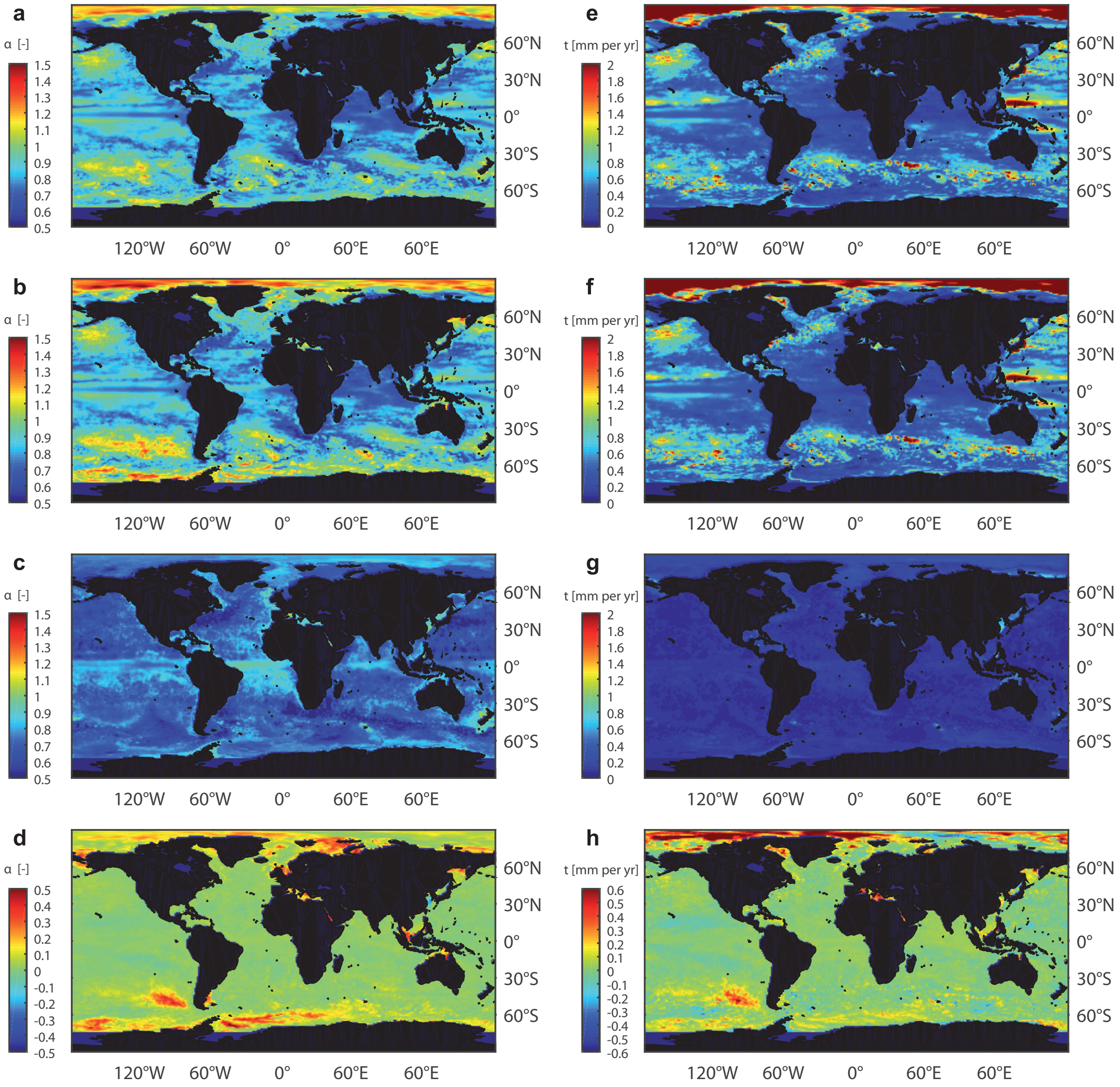

Supplementary Figure 6 | Hurst exponents α and maximum natural centennial trends in modelled LMSLsyn. **a-c**, α values as calculated for different components of LMSLsyn, RESsyn, and OBPsyn over the period from 1899 to 2008. **d**, Differences between the α values from LMSLsyn and RESsyn. **e-g**, Maximum natural trends (P=0.95) for LMSLsyn, RESsyn, and OBPsyn fields under the assumption of a short-term (α=0.5) or a long-term correlated (α>0.5) process. **h**, Differences between maximum natural trends in sea level derived from an integrated assessment of LMSLsyn minus the root sum of squares of natural trends calculated for OBPsyn and RESsyn separately.

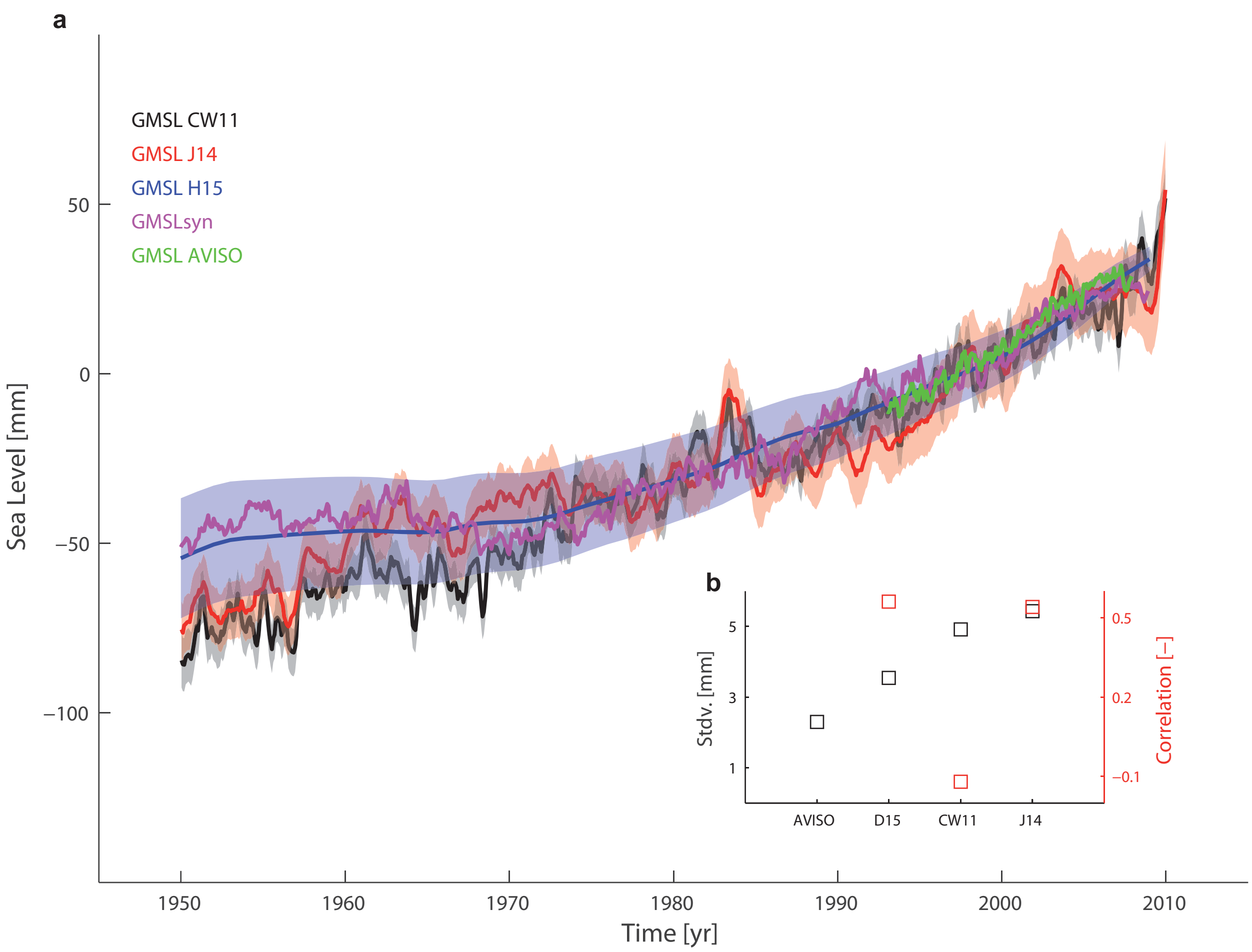

Supplementary Figure 7 | Comparison of different GMSL reconstructions and the GMSL from altimetry. **a**, Three different reconstructions from CW11[1] (black), J14[2] (red), and H15[3] (blue) in comparison to the true global mean from AVISO SSH[4] (green). Also shown is the modelled GMSLsyn curve from this study. The shaded areas show the respective 1σ uncertainties. **b**, Standard deviations (black squares) from detrended AVISO GMSL, GMSLsyn, CW11, and J14 over the period 1993-2008. Also shown are the correlations between detrended GMSLsyn, CW11, and J14 with detrended AVISO GMSL. The H15 curve is not compared, since it only represents a smoothed signal of the GMSL.

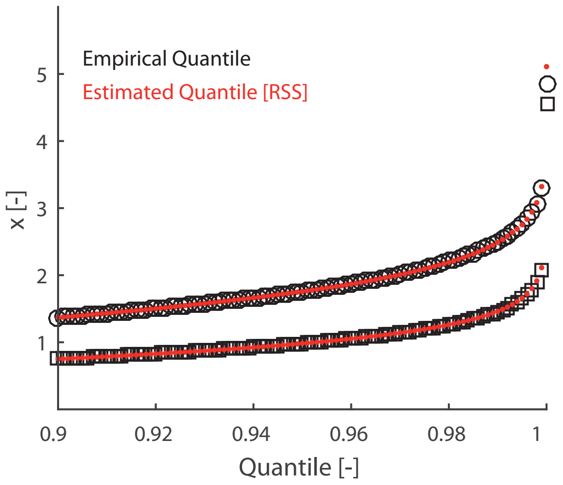

Supplementary Figure 8 | Performance of the root sum of squares (RSS) approximation for two independent/dependent t-distributed random variables. Shown is the comparison between the empirical quantiles of the sum of two t-distributed random variables (black) and quantile approximations using the root sum of squares (RSS, red) as described in equations 9 and 10 of the main document. The squares and dots in the upper curve correspond to the quantiles of independent random variables (equation 9 of the main document), while the circles and dots in the lower curve represent dependent random variables with a correlation of r = 0.5 (equation 10 of the main document).

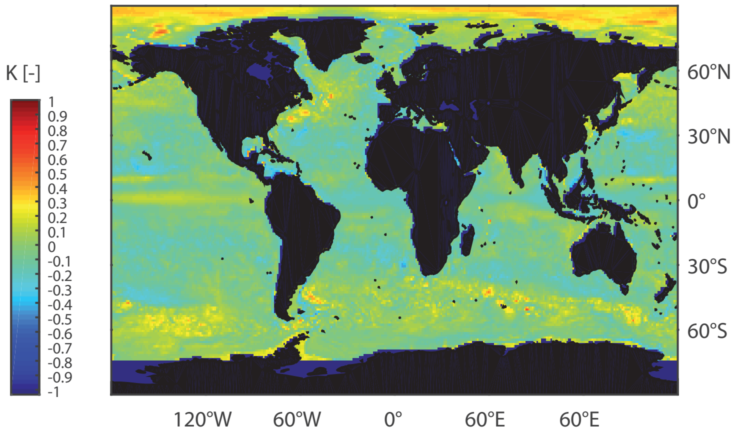

Supplementary Figure 9 | Correlations between RES and ATM. Shown are the linear correlations between RES and ATM. These are important for the summation of trend quantiles in equation 10 of the main document. It is clear that there are only a few regions with significant correlations between RES and ATM. Hence, their impact on equation 10 is only of minor importance in most regions.

Station	Availability	Hurst exponent α			LAG1 Autocorrelation c			Observed Trend [mm per yr] and Significance			Natural Trend [mm per yr]		External Trend [mm per yr]	
		**OBS**	**ATM**	**RES**	**OBS**	**ATM**	**RES**	**OBS**	**Case 1***	**Case 2****	**Case 1**	**Case 2**	**Case 1**	**Case 2**
Vlissingen	1900-2011	0,72	0,52	0,86	0,14	0,01	0,51	2,18	(1,00)	(1,00)	0,47	0,57	1,71	1,61
Hoek van Holland	1900-2011	0,71	0,51	0,86	0,17	0,00	0,45	2,24	(1,00)	(1,00)	0,50	0,58	1,74	1,65
Ijmuiden	1900-2011	0,73	0,52	0,90	0,16	0,01	0,51	2,04	(1,00)	(1,00)	0,61	0,75	1,43	1,29
Den Helder	1900-2011	0,67	0,54	0,89	0,13	0,06	0,45	1,25	(1,00)	(0,99)	0,45	0,64	0,79	0,61
Delfzijl	1900-2011	0,62	0,51	0,86	0,13	0,04	0,40	1,55	(1,00)	(0,99)	0,42	0,70	1,13	0,85
Norderney	1900-2011	0,61	0,56	0,84	0,15	0,11	0,50	1,84	(1,00)	(1,00)	0,43	0,65	1,42	1,20
Cuxhaven	1900-2011	0,60	0,56	0,82	0,16	0,11	0,50	1,76	(1,00)	(0,99)	0,46	0,61	1,30	1,16
Esbjerg	1900-2011	0,57	0,56	0,80	0,15	0,11	0,43	0,71	(1,00)	(0,81)	0,41	0,60	0,30	0,11
Hirtshals	1900-2011	0,65	0,56	0,86	0,20	0,14	0,37	1,17	(1,00)	(0,99)	0,49	0,74	0,68	0,43
Aberdeen	1900-2011	0,73	0,50	0,88	0,21	0,05	0,39	1,79	(1,00)	(1,00)	0,47	0,64	1,32	1,15
North Shields	1900-2011	0,88	0,52	0,96	0,35	0,04	0,53	2,29	(1,00)	(1,00)	0,89	1,02	1,40	1,27

*Case 1: Integrated Assessment

**Case 2: Separate Assessment

Supplementary Table 1 | Results from the estimation of natural and external trends in SLR. The table provides an overview over the main values derived from an assessment of each tide gauge record in the North Sea. The Hurst exponents α are derived from a DFA2. All trends are calculated for the common period from 1900-2011 using a confidence level of 95% (P=0.95). The significance S of each trend is provided in brackets.

Supplementary References

1. Church, J. A. & White, N. J. Sea-level rise from the late 19th to the early 21st century. S*urv. Geophys.*, **32**,585-602 (2011).
2. Jevrejeva, S., Moore, J. C., Grinsted, A. & Woodworth P. L. Recent global sea level acceleration started over 200 years ago? *Geophys. Res. Lett.*, ***35*(8)**, L08715 (2008).
3. Hay, C. C., Morrow, E., Kopp, R. E. & Mitrovica, J. X.. Probabilistic reanalysis of twentieth-century sea-level rise. *Nature.* **517,** 481-484 (2015).
4. Ducet, N. & Le Traon, P. Y. A comparison of surface kinetic energy and Reynolds stresses in the Gulf stream and the Kurisho systems from merged TOPEX/Poseidon and ERS-1/2 altimetric data. *J. Geophys. Res.*, **106**, 2671-2688 (2001).
